# Stricture of the Male Urethra

**Published:** 1877-02

**Authors:** Shirley Bragg

**Affiliations:** Lecturer on Urinal Surgery, Atlanta Medical College. Atlanta


ATLANTA

MEDICAL AND SURGICAL JOURNAL

VOL. XIV.] FEBRUARY—1877. [No. 11

### Original Communications.

### STRICTURE OF THE MALE URETHRA, WITH REPORT OF CASES.

BY SHIRLEY BRAGG, M.D.,
Lecturer on Urinal Surgery, Atlanta Medical College.

(*Read before Atlanta Academy of Medicine, January* 16, 1877.)

In reporting these cases it is not my purpose to write a didactic treatise on urethral stricture, but, as a prelude, will run hurriedly through the disease—diagnosis, treatment, etc.

Stricture may be defined to be an abnormal contraction, either spasmodic or organic; the former produced by irritability and spasm of the muscles of the urethra, the latter by the formation of "cicatricial tissue," following either chronic inflammation or traumatism. Aside from these, we come in daily contact with congenital contractions, constituting stricture, as of the meatus.

As organic stricture is the subject-matter of this paper, I shall pass over the spasmodic affection. Stricture, an abnormal contraction, caused by the formation of cicatricial tissue, as we generally find it, is produced by that most numerous of all urethral diseases, urethritis. Not only is it liable to follow a specific urethritis, but a non-specific. "A rupture—a blow to the organ may be productive of it; also loss of substance from urethral chancres, and ulceration from long continued pressure." It will not be necessary to enter into the minute anatomy of the parts, but merely to give the average length of the male urethra, which is from 8¼ to 8½ inches; its anatomical divisions and regions, viz.: the first or spongy division, the second or membranous, the third or prostatic. The spongy portion is the pendulous part of the penis, and extends from the bulb to the meatus, averaging $6\frac{1}{2}$ inches in length; the second or membranous portion extends posteriorly from the bulb to the prostate gland, measuring from $\frac{1}{2}$ to $\frac{3}{4}$ of an inch; the third or prostatic, extending from the membranous to the bladder, and is from 1 to $1\frac{1}{4}$ inches in length. The surgical regions are also three in number. The first region is at the sub-pubic curvature, or at the junction of the spongy with the membranous parts, including all the membranous portion, with an inch in front of it, making a length of $1\frac{3}{4}$ inches. The second region extends from within an inch of the membranous portion to within $2\frac{1}{2}$ inches of the external meatus, averaging in length from $2\frac{1}{2}$ to 3 inches. The third region is from $2\frac{1}{2}$ inches within to the external meatus. The location of the majority of strictures, according to the careful statistics of Thompson, found in 270 subjects, showing 320 strictures, are as follows:

Region 1 contained 215 strictures, or 67 per cent.

Region 2 contained 51 strictures, or 16 per cent.

Region 3 contained 54 strictures, or 17 per cent.

The frequency, then, of the majority of strictures at the first and third regions is perhaps "owing to the increased amount of erectile tissue and the greater vascularity of the parts." The appearance of stricture after gonorrhœa may be safely stated to be from one to twenty years; after urethral chancre a few months, owing to the higher grade of inflammatory actiou; and after an injury—traumatism—from a few months to several years, depending entirely upon the extent of violence, this including the rupture of a chordee.

In most cases there is only one stricture to be found; occasionally there are several contractions, but these are not always to be considered separate and distinct strictures, for we have a strictured condition sometimes of the whole spongy urethra, showing different degrees of contraction. Some urethral surgeons claim to have seen from three to six separate and distinct strictures; and one, Prof. Otis, of New York, claims to have seen *fourteen*.

There are few diseases more easily diagnosed than organic stricture. The French bougie, a boule or acorn-pointed bougie is the best instrument for this purpose we possess, not only to diagnose the distance of the stricture from the external meatus, but the length of the strictured part. Select your instrument (and they run up high in the scale) so that it will easily pass the meatus. After being well oiled, it is then carried slowly and carefully down the canal until its course is obobstructed. Then withdraw this slowly, and continue to pass smaller sizes, until one is found that will slide through the strictured point. This will give the extent of contraction. In withdrawing the instrument, place a finger upon its shaft at the meatus, measure the distance from the finger toward the bulbous end, and you will have the distance of the stricture from the meatus, thereby determining its exact locality. We also have another means of diagnosis but little used, viz.: by the endoscope.

In making a diagnosis of stricture, we sometimes come in contact with false passages, or with over-distended lacunæ, which may engage the point of our instrument, thereby causing it to leave its proper channel. Both care and skill are to be exercised here, else further injury is produced. In this instance we drop the bulbous bougies, for such passages are generally caused from the rough use of instruments accompanying tight strictures, and then resort to soft catheters or filiforms. After having diagnosed the strictured condition, carefully noted the general health of the patient, the treatment is determined.

Recognizing the gravity of the disease, numerous methods have been resorted to, not only to palliate, but to remove, as far as possible, the encroachments upon the normal canal. We have dilatation, gradual and continuous; divulsion and urethrotomy, internal or external. In olden times we had cauterization; although being weighed in the balance and found wanting in the treatment proper of stricture, it still holds a place in overcoming that irritability produced by late inflammatory action. Dilatation is handed down from time immemorial, almost, as standing pre-eminent in the treatment of this disease. Far beyond the sixteenth century instruments for dilatation are spoken of; in fact, excavations from the ruins of Pompeii demonstrate without a doubt the use of such in the early part of the Christian era.

Each and every one of these have their followers, and it is not my intention to discuss the various methods of treatment minutely, but only to put forth that treatment which in my hands has been the most brilliant. When we have the time, and strictures that are not dense to deal with, gradual dilatation is the treatment; but where we find strictures within an inch or two of the meatus, or in the whole spongy urethra, dilatation, nor any means devised, except urethrotomy, will not answer. Any operation, however, on the urethral canal must be followed, for either palliation or relief, by dilatation.

But the class of cases that we generally deal with are those who have been strictured for years, and which gradual pressure, owing to their extent and density, will not overcome. Divulsion and urethrotomy, in this class of cases, are the means left us. Divulsion is the introduction of a closed instrument through the stricture, expanding with a screw at the handle, thereby tearing or lacerating the parts. Urethrotomy is an incision of the cicatricial tissue.

Numerous instruments have been devised for performing internal urethrotomy, from the early part of this century to the present day; and, not to go over the long list of inventions, will only mention the instrument of Maisonneuve, as modified by Bumstead, of New York, for the incision of strictures of small caliber; and the late invention of Prof. Otis, which combines the divulsor and urethrotome, for those of large caliber. It is claimed by Prof. O. that in the use of his instrument the strictured condition is permanently relieved, but as yet it has not been sufficiently tested. For the *relief* of this disease, external urethrotomy gives the best results. By this operation we can divide every cicatricial band thoroughly; whereas, in internal urethrotomy and divulsion we cut or tear sufficiently to gain entrance into the bladder, not knowing, and having no means of knowing, whether or not the whole strictured mass is divided; and if ever an organic stricture is entirely and permanently relieved, it will be by external urethrotomy, which will never fail to give more brilliant results (if properly performed) than any operation yet devised. All urethrotomes, however well they may be guarded, wound to a certainty in many instances the healthy mucous membrane. For the internal division of a stricture Otis' instrument is the best. It too, however, is liable to this fault. But why is not external urethrotomy more frequently performed? Chiefly owing to the gravity of the operation, but sometimes to the fears of your patient; whereas the internal, when properly done, is devoid of any very great danger. When there is a dense stricture, one or the other of these operations I do; but when we have the time, and stricture that will yield to dilatation, I resort to this method. Divulsion shows some good results, but Mr. Thompson, who is the inventor of the instrument bearing his name, says that urethrotomy gives better.

I have used this instrument (divulsor) but little, and in my opinion it is better either for gradual dilatation or to precede a urethrotome. Surgeons agree that it is safer for strictures in the region of the bulb, on account of its great vascularity, and as I am almost a partisan to urethrotomy, can safely say I have never seen such hemorrhage following the operation. But in performing these operations there are other points to weigh. Never touch a patient without first examining his urine and looking carefully to his every symptom; for when there is need for an operation, generally there is more or less constitutional disturbance, all of which must be properly met. In my operations, quinia and iron, for days before the operation, are administered and kept up. When these have been used rigors have been seldom, and urethral fever, if any, slight. After preparing the patient in this manner, his system is in a fit condition to withstand the troubles that might arise.

The steps of the operation for internal urethrotomy will be given briefly. After placing your patient on a firm, hard bed or table, with the knees drawn up, giving or not giving an anæsthetic, the penis is first filled with oil; then a delicate filiform is passed in, either of rubber or whalebone, until its course is obstructed; another and another is carried down, until one strikes the entrance, thus giving control of the bladder. The urethrotome is then attached to this filiform, after the others are withdrawn, which serves to guide it through the strictured part. The knife is now introduced, and as free a division made of the cicatricial tissue as possible. After the operation a full-sized sound is carried in. If the bladder contains urine draw it off. Forty or fifty drops McMunn's elixir opii, with ten grains quinia given, and if there is much oozing of blood, the application of ice to the penis. The patient is then instructed to hold his water as long as possible. It is drawn off for twenty-four hours; after this is allowed to pass in a tub of hot water. In three, four, or five days the sound is again passed, beginning with two sizes smaller than when you last left off, so as to gradually distend the canal, and in a great measure to obviate the pain caused by the introduction of a full-sized sound. But not to go further, I herewith append a few cases, showing manner of treatment, results, etc.

Cicero (col'd) was sent to me by my friend, Dr. L., of this city, December 7th inst. Upon examination found him suffering with a constricted meatus and two strictures; one just in front of the scrotum, the other at the bulb; also two fistulous openings, one in the perineum, the other to the left side, both of which allowed the passage of urine. He stated that he had been in this condition five years, and losing flesh daily. Was a perfect wreck, with little or no hope, complaining of rigors, and, to all appearances, kidney troubles had loomed up. I made a careful examination of his urine, both chemically and microscopically, and after determining the condition of his kidneys, concluded to operate by divulsion, with a view to breaking up the strictured mass, and as there was no appreciable loss of substance, to heal the fistulous openings. Accordingly, on the 10th December, after preparing his system, the operation was performed. After a considerable length of time a whalebone guide was passed. I then proceeded, first by incising the meatus, giving me full control of the lower canal. After doing this Thompson's divulsor was introduced over the filiform; the blades rapidly distended, and the operation was completed without the administration of an anæsthetic. After withdrawing the divulsor, No. 25 F. scale was passed; a full dose McMunn's elixir opii and quinia administered, and the patient instructed to hold his water as long as possible.

Saw him late that evening. Water was drawn off. Next morning was allowed to pass it in tub of hot water. Third day after operation patient was out of bed; had had no fever or bad symptom. On the sixth day the urine ceased to pass through the fistules, and in one month's time they had disappeared. He was told to come to my office every fourth or fifth day, but being of a timid disposition, and finding he could pass his water with all ease, did not make his appearance.

Have seen him several times since. Says he is in fine health and experiences no trouble. His appearance corroborates this. Owing to his failure to come for the passage of the instrument, there is naturally more or less recontraction, which in course of time will cause him again to seek the aid of a surgeon.

J. S. P., of Atlanta, consulted me in the latter part of December, 1875, for what he considered a kidney trouble. Had suffered with his back for years; also telling me that he was subject to rheumatism, and the year before had had violent cystitis, for which his bladder was injected daily. Examined his urine carefully, both chemically and microscopically; found it normal, with the exception of a great amount of pus. I told him to call the next day for examination, which he did When just beginning to make the examination he complained of a dull pain in the perineum and back; also a sense of uneasiness about the bladder, with a constant desire to urinate, though he had passed his water but a few minutes before. As expected, I found a very narrow meatus, and on exploration with a bougie a boule discovered two and a half inches in a distinct stricture. The canal was examined in its whole course, and no other contractions found. The prostate was also examined carefully, and found to be, in a man of his age, normal.

Suggested the propriety of an operation, to which he readily consented, as he would have done to anything, having been such a martyr to pain. Accordingly he was put on full doses of quinia and iron, with a cit. potass. mixture to render the urine bland.

January 15th, 1876, the operation was performed, by incising first the meatus, then the deeper stricture. No anæsthetic was administered, and hemorrhage was slight. No. 25 F. scale was passed with all ease; a full dose elixir opii and quinia given. Having him so thoroughly under the influence of quinia, he was allowed to pass his urine from the beginning. Was out of bed the second day, coming regularly for the introduction of an instrument every third or fourth day, until No. 30 F. scale was passed. Four or five days, however, after the operation there was considerable irritation. Prescribed aqueous ex. opii ℈j, aqua ℥iv; inject after urinating.

January 30, was discharged entirely relieved, with instructions to purchase an instrument and pass it once a month, which he continues to do. See him daily; the constant desire to urinate is entirely relieved, and suffers no trouble from the perineum or back. Complained of having also been impotent. This is also relieved, and the organ restored to its natural powers.

Mr. J. R. M., of Southwest Georgia, applied May 20, 1876, for consultation, stating that he was suffering from some urinary trouble. At times was very comfortable, but when the least imprudent his urethra would become greatly irritated. Had some year or two before contracted a gonorrhœa, which had been cured by injections of nitrate silver, but returned several times after traveling or riding horseback. Since that time had tried injections and internal remedies more numerous than he could mention, without any perceptible benefit. I suggested an examination. Found the meatus constricted; two inches deeper a dense stricture, from the rupture of a chordee, and just in front of the bulb another, admitting only the smallest filiform.

Explained to him that an operation was necessary, to which he at last consented. After placing him on the same treatment referred to previously, determined to operate May 25th, in the presence of Dr. H. B. L., of this city, who kindly consented to administer chloroform. The patient was a bad subject for operation, having had chills, and in fact only one or two weeks out of a malarial climate. However, wanted the operation done immediately. The anæsthetic being administered, the urethra was filled with filiforms, until one was found to pass. The others were then slowly withdrawn. The meatus was first thoroughly incised, Maisonneuve's instrument attached, and both strictures partially divided. After withdrawing this, Otis' urethrotome was introduced, expanded to the normal calibre, according to measurement, and the cicatricial tissue incised to admit No. 25 F. scale, with the view of producing, as he claims, a radical cure; and up to this time the results are highly satisfactory. After the operation, one-half grain morphia, with ten grains quinia, was administered. Fearing some trouble from the kidneys, he was put on a mixture containing thirty grains cit. potass. to the dose; tablespoonful every two hours; pounded ice was also applied to the penis and perineum.

Called to see him late that evening. Found him doing well; relieved the bladder by catheter. After resting comfortably, allowed him to pass his urine next morning in a tub of hot water. Out of bed third day. Full-sized sound was introduced every fourth day, until the parts were healed.

After operation had slight fever, which yielded to proper treatment. Had no rigor, though of a nervous temperament. Was supplied with a proper sized instrument and discharged in fine health. Examined him several times after the operation, and could find no contraction in any part of the canal.

Jim —— (col'd) was sent to me July 25th, by Dr. O., of this city. Found him with phymosis (congenital), which was immediately removed, the wound healing by granulation; was also passing blood in large quantities, and was so weak and emaciated as to be confined to bed.

The meatus was constricted, the penis feeling like bone, and upon exploration found the urethra literally strictured from one end to the other, showing different degrees of contraction. This case, according to the views of Prof. Otis, presented twelve or fifteen distinct strictures. He agreed to submit to an operation. Found but little difficulty in introducing the smallest filiform, but an impossibility to follow it with any instrument, owing to the tortuous course of the stricture. I then concluded to try continuous dilatation; accordingly tied the filiform in, as it did not interfere, to any great extent, with the passage of urine, for thirty-six-hours. At the expiration of this time Otis' urethrotome was introduced over the guide, the instrument expanded to the normal caliber, and the whole strictured mass divided so as to admit No. 27 F. scale. Done without anæsthetic. After-treatment, opium, quinia, and cit. potass. mixture. Urine drawn off for twenty-four hours, then allowed to pass it in tub of warm water. No fever nor bad symptoms until fifth day, when an abscess began to form in the perineum; in fact, was threatened with this before operation. Made an incision in the median line, almost down to the urethra, without finding pus, and with only temporary relief. In a few days, finding my patient losing strength, made the incision deeper, finding pus and going into the urethral canal. Patient was immediately relieved. The passage of the sound was kept up, the parts healing kindly. Twenty-eight days after operation urine is clear; appetite good, and sleeps well. In my office a few days since; No. 27 F. scale passed with all ease. The penis feels natural, and the patient in good health.

Mr. T. M. H. Was called to see this gentleman by my friend, Dr. O. Found him suffering very much from retention of urine. Told me that he had a stricture of twenty years standing, but up to that time his health had been good. Had been operated on before the war. In making an effort to withdraw his water, found the canal filled with false passages; after time and patience, however, succeeded in introducing the smallest catheter. After removing catheter it was impossible to introduce a filiform. Put him on quinia and iron; trying every day or two to introduce a filiform, in which I was unsuccessful. Knowing that retention was liable to re-occur at any moment, determined to do external urethrotomy. The patient consented, and on July 26th the operation was done; Dr. P., of this city, kindly administering the anæsthetic. The patient was placed in the position for lithotomy, on a hard bed. The stricture was impervious to the passage of any instrument. A grooved staff (the one used by Syme) was introduced as far as possible, and pressed firmly against the stricture. An incision was then made extending from the back part of the scrotum to near the anus, exactly in the median line along the raphe. The urethra was opened on the point of the instrument in front of the stricture. Search was made for the posterior opening, which with some difficulty was found. A grooved director was then let into the bladder, and upon this the opening was enlarged so as to admit No. 29 F. scale. After the operation opium and quinia were administered, and the wound plugged with lint to prevent hemorrhage. There was some shock, from which he soon recovered. Was allowed to pass his water, which for several days came through the wound. No catheter was tied in. He went to New York in a few weeks, gaining both strength and flesh. Owing to sickness of myself he did not return, as directed, for the passage of an instrument. He passes his water freely, and up to the time of a fall some week or so ago, was in good health. He had no fever, and no bad symptoms further than previously mentioned, though these were anticipated on account of his age, his health, and the long-standing stricture.

These cases are but imperfectly reported; just from notes taken at the time; hence are not in all respects as elaborate as I should like them.

